# Automated Detection of Broncho-Arterial Pairs Using CT Scans Employing Different Approaches to Classify Lung Diseases

**DOI:** 10.3390/biomedicines11010133

**Published:** 2023-01-05

**Authors:** Sami Azam, A.K.M. Rakibul Haque Rafid, Sidratul Montaha, Asif Karim, Mirjam Jonkman, Friso De Boer

**Affiliations:** 1 Faculty of Science and Technology, Charles Darwin University, Darwin 0909, Australia; 2 Health Informatics Research Laboratory (HIRL), Department of Computer Science and Engineering, Daffodil International University, Dhaka 1216, Bangladesh

**Keywords:** COVID-19, chest CT scan, lung segmentation, image preprocessing, CNN, LSTM, attention mechanism

## Abstract

Current research indicates that for the identification of lung disorders, comprising pneumonia and COVID-19, structural distortions of bronchi and arteries (BA) should be taken into account. CT scans are an effective modality to detect lung anomalies. However, anomalies in bronchi and arteries can be difficult to detect. Therefore, in this study, alterations of bronchi and arteries are considered in the classification of lung diseases. Four approaches to highlight these are introduced: (a) a Hessian-based approach, (b) a region-growing algorithm, (c) a clustering-based approach, and (d) a color-coding-based approach. Prior to this, the lungs are segmented, employing several image preprocessing algorithms. The utilized COVID-19 Lung CT scan dataset contains three classes named Non-COVID, COVID, and community-acquired pneumonia, having 6983, 7593, and 2618 samples, respectively. To classify the CT scans into three classes, two deep learning architectures, (a) a convolutional neural network (CNN) and (b) a CNN with long short-term memory (LSTM) and an attention mechanism, are considered. Both these models are trained with the four datasets achieved from the four approaches. Results show that the CNN model achieved test accuracies of 88.52%, 87.14%, 92.36%, and 95.84% for the Hessian, the region-growing, the color-coding, and the clustering-based approaches, respectively. The CNN with LSTM and an attention mechanism model results in an increase in overall accuracy for all approaches with an 89.61%, 88.28%, 94.61%, and 97.12% test accuracy for the Hessian, region-growing, color-coding, and clustering-based approaches, respectively. To assess overfitting, the accuracy and loss curves and k-fold cross-validation technique are employed. The Hessian-based and region-growing algorithm-based approaches produced nearly equivalent outcomes. Our proposed method outperforms state-of-the-art studies, indicating that it may be worthwhile to pay more attention to BA features in lung disease classification based on CT images.

## 1. Introduction

Computed tomography (CT) is commonly used for the interpretation of lung diseases as the modality can provide indispensable information regarding airways as well as the blood vessels [1]. Changes in broncho-arterial pairs (BA), such as abnormal bronchial dilatation compared to the adjacent artery, distortion of the bronchial tree, lack of tapering, mucus plugging, air bronchogram, and airway wall thickening, are associated with the progression of lung disorders [2]. These deviations and features can be subtle and difficult to perceive with the human eye. Deep learning techniques might lead to improved results if these BA features are enhanced and highlighted. Therefore, changes in BA in terms of structure or features should be taken into account in the diagnosis of lung abnormalities. Several lung disease detection and classification studies have been conducted over the years. In many cases, lung segmentation, image preprocessing, feature extraction, deep learning, or machine learning methods have been applied [3]. However, selection bias, prospective blinding concerns, and the confounding presence of different lung diseases in the same chest CT scan [4] limit their usefulness. In lung diseases such as bronchiectasis and airway inflammation [5], the quantitative measurement of the BA ratio using the long-axis diameter and short-axis diameter of the bronchus and artery [6] is used as a severity score which is an indication of disease progression. For the diagnosis of cystic fibrosis (CF), asthma and smoking-related lung disorders, and pediatric diffuse lung disorders, BA ratio measurements and airway wall thickening are also analyzed [7,8,9]. For other lung diseases, BA feature alteration can also be considered an important factor. Structural distortion of BA, the BA ratio, the thickness of the airway wall, and mucus plugging should be examined in severe stages of COVID-19 and pneumonia [10]. Automated detection of BA deviations in CT scans might be useful in the classification of lung disorders. In this research, we therefore focus on the enhancement of BA and highlighting of BA irregularities using different computer vision techniques. A CT Scan dataset of three classes, named Non-COVID, COVID, and CAP (community-acquired pneumonia), containing 17,104 images, is used. The dataset is preprocessed, and the lungs are segmented from the CT scan images. BA information is enhanced and highlighted using four approaches: Hessian-based approach, region-growing algorithm-based approach, clustering approach, and color-coding approach. Hence, four new datasets are generated. To find the optimal approach, diseases are classified using two deep learning mechanisms: (a) a convolutional neural network (CNN) and (b) a CNN with long short-term memory (LSTM) and an attention mechanism, employing the four processed datasets. During the implementation stage of this study, lung segmentation and enhancement are carried out to ensure maximal visibility of bronchi and arteries, as this may increase the performance of the classification model. In the segmentation phase, the region of the lungs is selected as this is the important region of the CT scan. Accurate lung segmentation for all images is quite challenging due to different intensity levels. We address this using different algorithms and a dynamic thresholding approach. It is challenging to enhance the BA pairs while keeping other information about the lungs intact. Therefore, four approaches are explored. The Hessian and region-growing-based algorithms are applied in such a way that all the bright regions that contain arteries and potential bronchi are emphasized. The color-coding and clustering approaches ensure that the output enhanced images contain as many relevant details as possible. Another challenge is to develop a shallow model while maximizing the performance. Various ablation studies are done in developing the architecture to achieve the best possible configuration for this classification problem and obtain the maximal accuracy. The model is compact, containing as few layers as possible to minimize the number of parameters and reduce the complexity.

## 2. Materials and Method

### 2.1. Prior Work

In a study of bronchiectasis in children [11], the BA ratio of progenies was compared with the radiologic BA ratio criteria of adults using high-resolution CT (HRCT) images. The BA ratio was measured for both the upper and the lower lobe, and the mean value was calculated to determine the correlation with age. In a similar study [12], the authors suggested a threshold value; >0.8 should be used to conclude bronchiectasis, whereas the adolescent cut-off is >1–1.5. Here, the value for the BA ratio is determined by measuring the central diameter of each of the bronchus and the external diameter of the accompanying artery. In order to diagnose coronary artery disease (CAD), Zhao et al. [13] segmented coronary arteries using a multi-input multi-scale U-Net model along with different preprocessing strategies and computed the diameter of the vessels. Chang et al. [14] presented an HRCT scoring system to diagnose cystic fibrosis (CF) bronchiectasis following three different schemes for children of different ages. Among the cases they investigated, a former history of pneumonia was found for all children where an abnormality was detected. To define the diagnostic criteria of pediatric bronchiectasis, Wu et al. [6] obtained the BA ratio from CT scans using three techniques: bronchial internal short axis (ISA), outer short axis (OSA), internal long axis (ILA), outer long axis (OLA), arterial long-axis (ALA) and arterial short axis (ASA). Several statistical metrics, including intraclass correlation (ICC), mean, skewness, kurtosis, and Pearson’s correlation, were evaluated. In order to develop a CT grading system for the diagnosis of cystic fibrosis (CF), Bhalia et al. [15] considered several pathologic deviations, such as bronchiectasis, peribronchial condensing, mucus plugging, luminal dimensions, and thickness of bronchial walls. In another study [16], the radiologically indexed CT Score was determined using HRCT for the severity of bronchiectasis depending on a multi-variable Bhalla score, and its suitability for medical practice was discussed. The Bhalla score was related to the severity indicators, including dilation of bronchi and quantity of bronco-pulmonary fragments with emphysema, in a multiple logistic regression network. Using HRCT, an automated broncho-vascular pair (BV pairs) system was presented by Prasad et al. [17], who explored several techniques, including multiview learning adjoined with active learning, feature extraction, and a decision tree algorithm. The authors first detected all the BV pairs, then extracted only the discrete pairs and identified abnormal pairs and the degree of severity.

Several studies have described changes in BA features related to COVID-19 and pneumonia. In the study of [18], seven patients having both COVID-19 and bronchial artery embolization (BAE) were investigated. Three patients were reported with bronchial artery enlargement, and three patients had consolidation. To categorize vessels into veins and arteries using CT mages, Nardelli et al. [19] introduced several automated deep learning techniques, including 2D and 3D CNNs. A graph-cut-based approach recorded the highest accuracy of 94%, outperforming CNN and random forest methods. An automated pulmonary vessel segmentation from CT images approach was presented by Zhou et al. [20]. Several algorithms were carried out consecutively, including the 3D multiscale filtering technique, eigenvalues of the Hessian matrix, expectation-maximization (EM) estimation, and connected-component analysis to extract and reconstruct the vascular structures. They achieved the highest accuracy of 97% in segmentation and reported that in many cases (>96%), arteries overlap with vessels. Ambrosetti et al. [10] investigated two COVID-19 cases in order to find whether bronchiectasis (bronchial dilation) is a potential imaging feature of COVID-19. In both cases, they found dilatation and irregular walls of the bronchi leading to bronchiectasis. Hence, their suggestion is to take bronchial features into account in the interpretation of COVID-19 and pneumonia CT scans. Examining CT scans of COVID-19-positive cases, Hefeda et al. [21] found several structural distortions associated with bronchi and arteries. Anomalies include air bronchogram, vascular dilatation, bronchial wall thickening, and bronchial obstruction. Gu et al. [22] reported that several structural and functional alterations, such as distortion of the main bronchial tree, reduction of the bronchial cross-sectional region, and airflow obstruction, can occur due to lung cancer.

It can be concluded that an association between BA and lung disorders such as COVID-19 and pneumonia has been reported and that broncho-arterial pair detection may be relevant for these diseases. Nevertheless, to the best of our knowledge, there has been no research describing an automated system to detect anatomical and structural distortions of BA due to COVID-19 or pneumonia.

### 2.2. Research Contribution

- The anomalies and alteration of BA information in a chest CT scan dataset, such as abnormal bronchial dilatation, distortion of the bronchial tree, lack of tapering, mucus plugging, air bronchogram, and airway wall thickening, are briefly explained with respect to the different lung diseases.
- Several image preprocessing and lung segmentation steps, including total variation denoising, dynamic intensity adjustment, Otsu thresholding, largest contour detection, inverting image, flood fill operation, inner hole filling, and bitwise_AND, are introduced.
- In the segmented lung regions, BA information is enhanced and highlighted using four approaches: Hessian-based approach, region-growing algorithm-based approach, clustering approach, and color-coding approach.
- Lung diseases are classified for the four processed datasets (Hessian, region growing, clustering, and color) using two deep learning models. The first is a CNN model having 14 layers, and the second is a CNN-based model with LSTM and an attention mechanism, having 16 layers.

### 2.3. Dataset

A total of 17,104 lung CT scan images provided by Kaggle were analyzed for this research [23]. The dataset was divided into three classes named Non-COVID, COVID, and CAP, where 6983 lung CT images are Non-COVID, 7593 images are COVID, and 2618 are CAP. The images have a 512 × 512 pixel size. A description of the dataset is given in Table 1.

**Figure 1:**
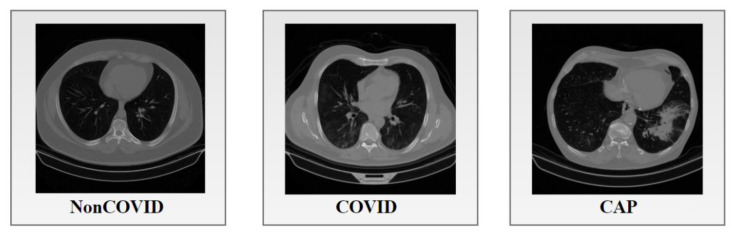
CT images of the three classes of the dataset.

A sample image of each corresponding class is illustrated in Figure 1.

### 2.4. Anatomical Features of Lung CT Scans

**Figure 2:**
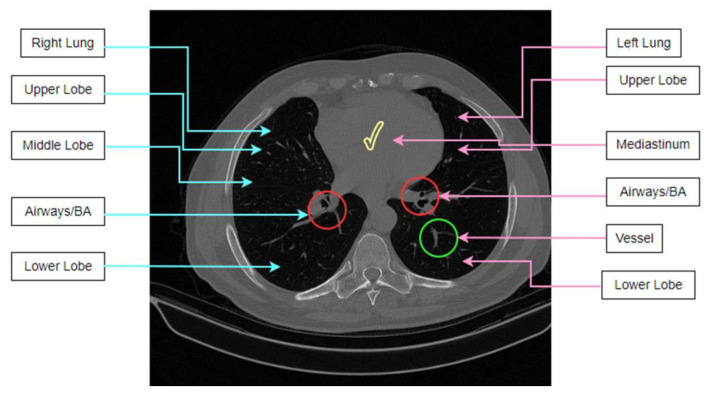
Anatomical structures in a lung CT scan.

A CT scan contains information regarding the airways and blood vessels which can yield essential information for diagnosis, e.g., the diameter of arteries and bronchi and the extent of wall thickening. For an automated and reliable measurement system of these changes, knowledge regarding the anatomical structures visible in a CT scan is crucial. Prior studies have not taken bronchi-artery alterations into account. As this research attempts to consider these alterations in classifying lung diseases, it is important to understand the anatomical features of lung CT scans. Figure 2 shows the anatomical structures of a lung CT scan.

The human body has two lungs, with the mediastinum between them. These can be seen in a CT scan. Both lungs contain veins, arteries, and BA pairs. The lungs are subdivided into lobes, where the left lung comprises two distinct lobes, the upper and the lower lobes, and the right lung part has three lobes, the upper, middle, and lower lobes. The airway tree is called the bronchial tree. The starting point or first branch of the tree is the trachea. The trachea is divided into two prominent bronchi leading to the left lung and the right lung. These two main bronchi are then subdivided into several smaller bronchi. The airways comprise lumen and airway walls where the lumen is normally filled with air. Moreover, two vascular trees are found, the arterial and venous tree, where the arterial tree is responsible for supplying blood to the lungs, and the veins are responsible for draining blood from the lungs. The veins and arteries have similar intensity levels in CT images. One important aspect is that arteries are usually observed to be adjacent to the bronchi, whereas the veins typically do not run alongside the bronchi.

#### 2.4.1. Anomalies Related to Lung Disease

Lung abnormalities include abnormal bronchial dilatation, distortion of the bronchial tree, lack of tapering, mucus plugging, and airway wall thickening. These are associated with lung disorders. This section explains these abnormalities and changes.

##### Bronchial Dilatation

**Figure 3:**
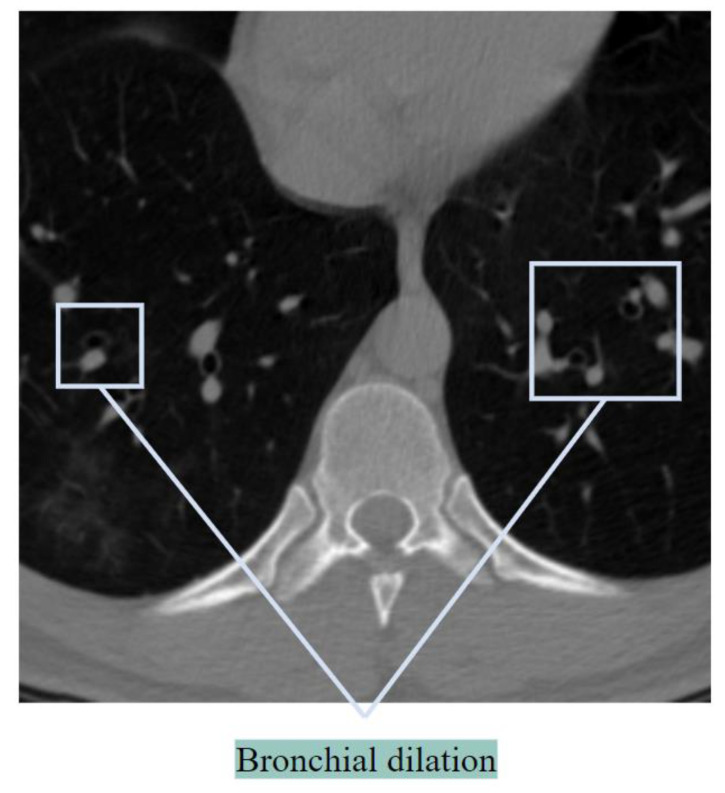
Bronchial dilation showed in a CT scan.

In healthy lungs, the diameter of a bronchus is either equal to or slightly smaller than the diameter of its neighboring artery. When the bronchus is considerably larger than the size of the accompanying artery, this is called bronchial dilation, and BA pairs can look like a signet ring [17]. Figure 3 illustrates the bronchial dilation.

If Figure 3 is carefully observed, it can be seen that the BA ratio (ratio of the diameter of the bronchus to its accompanying artery) is increased, and the BA pair can be perceived as a signet ring sign. The challenge is the detection of discrete BA pairs having a signet ring shape. In many cases, a bronchus is close to multiple arteries, or several bronchi may appear together, which complicates the matter. Bronchial dilation has also been detected in some COVID-19 cases, with a BA ratio that was almost doubled [10]. In the case of pneumonia, dilatations occur along with the destruction of bronchi.

##### Distortion of the Bronchial Tree

Distortion of the bronchial tree occurs in several forms, such as a decline of the angle between the trachea and the left major bronchus, a sigmoidal deformation of a major bronchus, and stenosis of the lower lobar bronchus can be found after lobotomy due to lung cancer [22].

##### Lack of Bronchial Tapering

**Figure 4:**
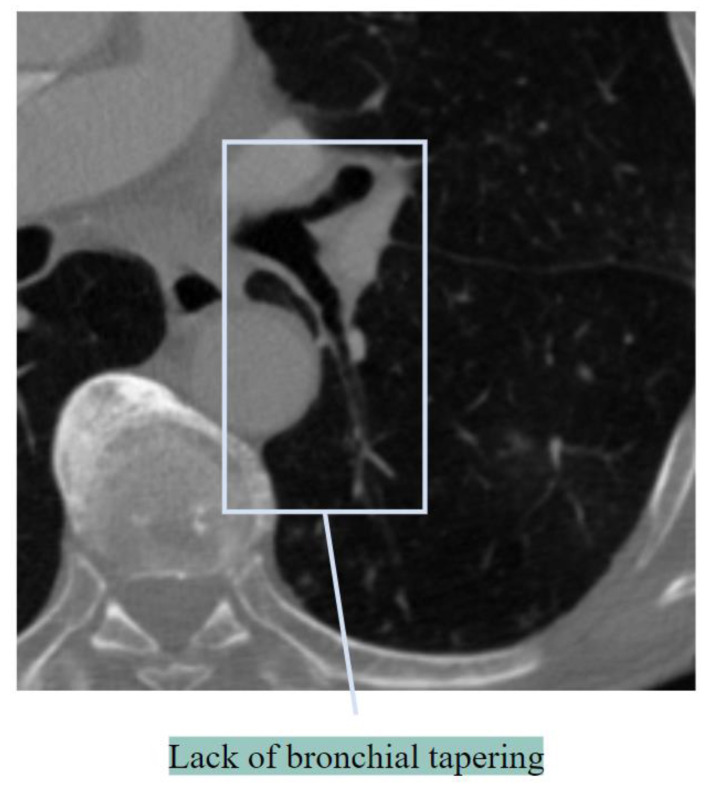
Lack of bronchial tapering.

Lack of tapering refers to a lack of change in the airway diameter by 2 cm [24]. This results in an increased BA ratio. Figure 4 illustrates a lack of tapering.

##### Airway Wall Thickening

**Figure 5:**
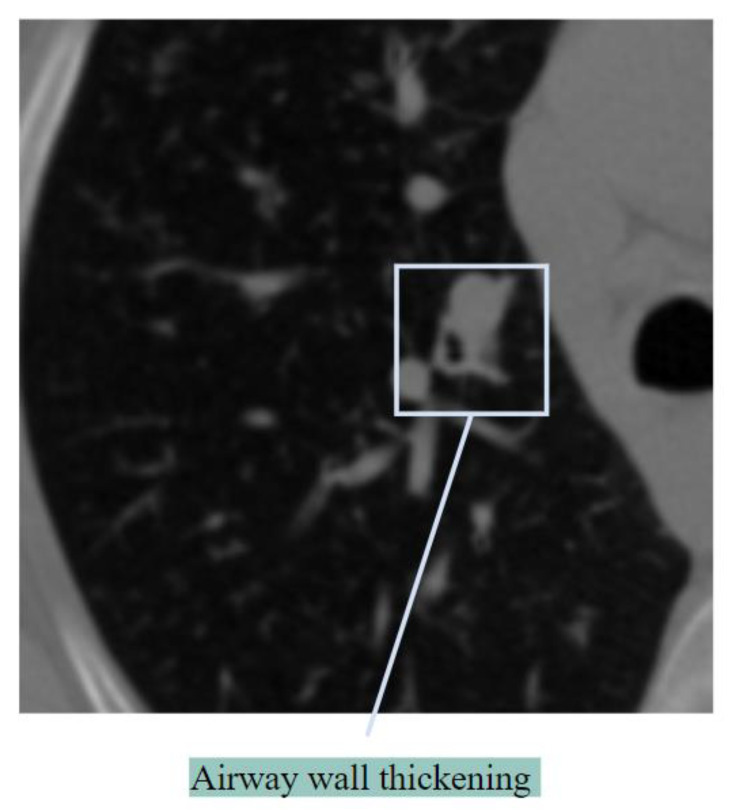
Airway wall thickening.

Bronchial wall thickening, which can result in airflow blockage, is another indicator of lung abnormalities [25]. Airway wall thickening generally occurs in inflammation of the airways. Figure 5 shows airway wall thickening.

Two pathological mechanisms, bronchial obstruction and airway wall inflammatory destruction, result in damage to bronchial wall structures [26]. Bronchial wall thickening is also associated with severe cases of COVID-19.

##### Air Bronchogram

**Figure 6:**
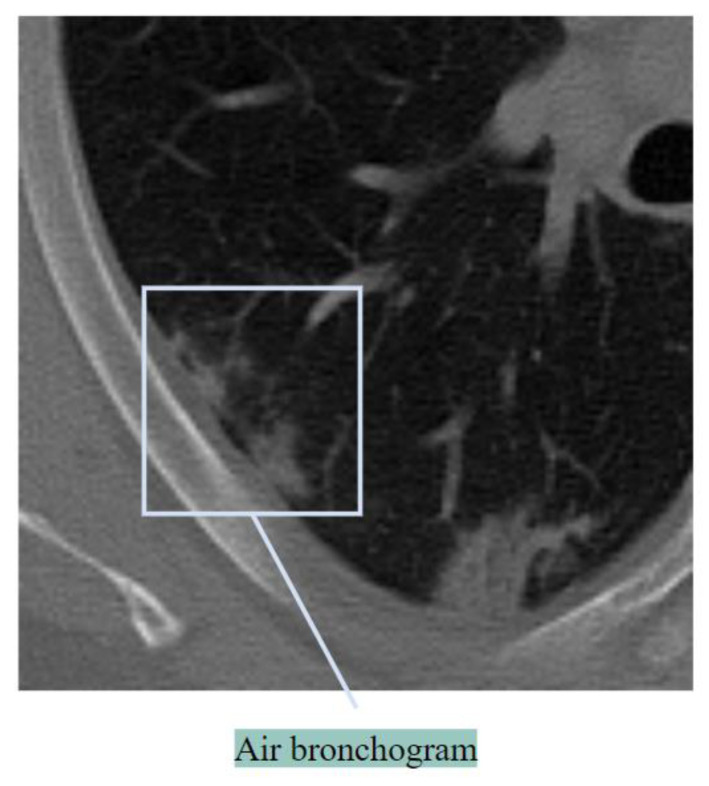
Air bronchogram.

An air bronchogram refers to a bronchus that is excessively filled with air. This feature is an indication of advanced disease [21]. Figure 6 shows air bronchogram regions of an abnormal lung.

An air bronchogram can be an indication of lung disorders like tuberculosis and pneumonia [27]. In classifying lung cancer, air bronchogram can also be important. An effective process of highlighting these anomalies should be considered.

##### Mucus Plugging

**Figure 7:**
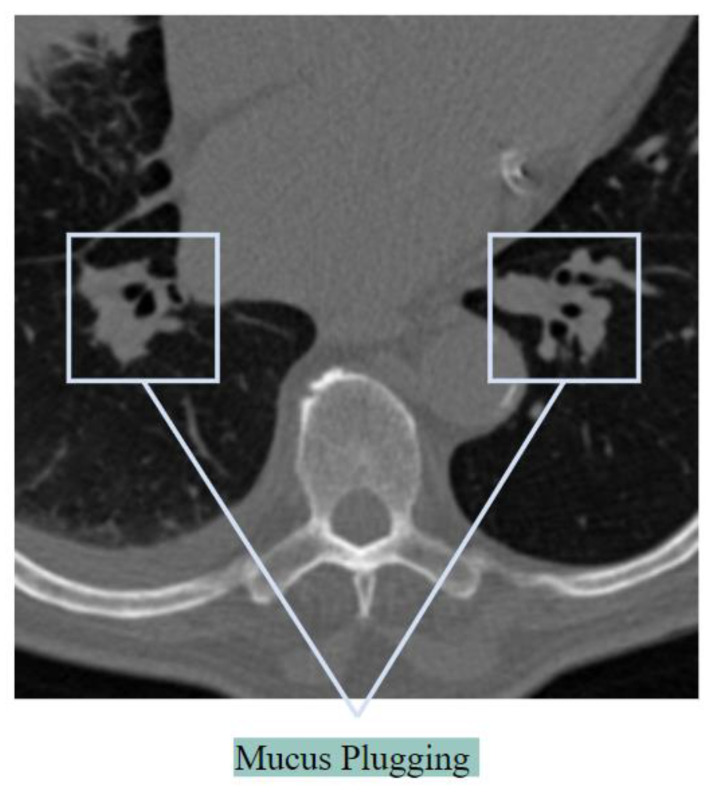
Mucus plugging as seen in a CT scan.

The previously stated word ‘air bronchogram’ is sometimes regarded as incorrect as the bronchi are sometimes full of plugs of mucus, not with the air [28]. Mucus plugs in the bronchi cause a decrease in the bronchial diameter, impeding airflow [29]. Figure 7 illustrates mucus plugging.

Mucus that builds up in the lungs might plug up, or decrease airflow in, the greater or lesser airways. As the airways become smaller due to mucus plugs, it causes distorted alveoli. When a considerable number of alveoli are blocked, oxygen levels will decrease. Thick mucus in the bronchi may cause symptoms like a dry cough in patients having COVID-19 [30].

### 2.5. Methodology

**Figure 8:**
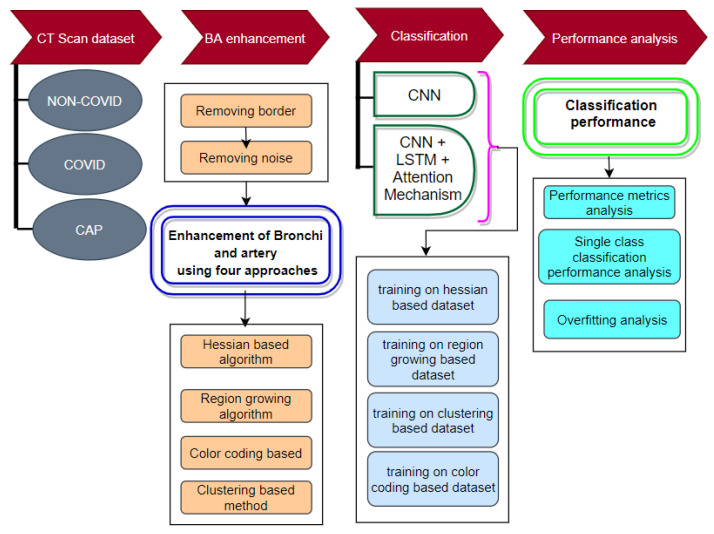
Flow diagram of the approach of this paper.

This research is conducted in six phases: (a) segmentation of the lung area from the CT scan; (b) enhancement of BA pairs in the segmented lungs using a Hessian-based approach; (c) enhancement of BA pairs using a region-growing algorithm-based approach; (d) enhancement of BA pairs using a clustering approach; (e) enhancement of BA pairs using a color-coding approach; and (f) classification of lung diseases using the four processed datasets. Figure 8 depicts the workflow of this research.

All the experiments of this study are conducted using a CT scan dataset which has three classes: COVID, Non-COVID, and CAP. Several image preprocessing algorithms, such as the total variation denoising method [31], dynamic intensity adjustment, Otsu thresholding [32], largest contour detection [33,34], inverting image color, flood fill operation [35], inner hole filling, and bitwise_AND operation are applied to segment the lungs. After segmenting the lung regions successfully, the BA pairs are highlighted and extracted using four approaches: a Hessian-based algorithm [36], region-growing algorithm [37], clustering-based [38], and color-coding-based. Prior to this, adjacent borders of the segmented images and noise are removed. In the classification phase, two deep learning approaches are adopted: CNN [39] based classification and CNN with LSTM and attention mechanism-based classification. All four processed datasets are employed to train the networks. To assess the performance and stability, a number of performance metrics and statistical metrics, single class classification results and overfitting analysis, and k-fold cross-validation of these two models are evaluated.

#### 2.5.1. Lung Segmentation

**Figure 9:**
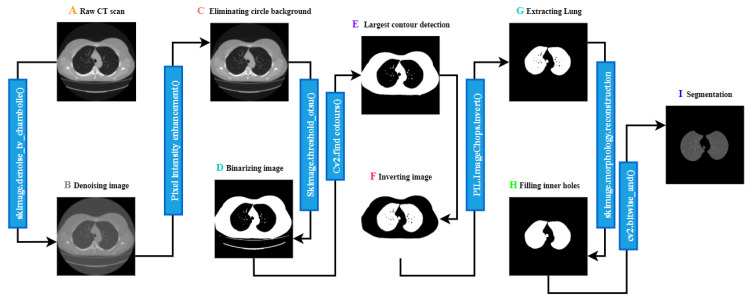
Lung segmentation process.

To identify abnormalities, the first stage should be to identify and extract the lung portion of the CT scans. This is the region of interest (ROI) for this research. For the computerized interpretation of medical images, segmentation is usually considered imperative [40]. The raw CT scan images are denoised employing the total variation denoising method, which eliminates noise, focusing on edge details. The circular shaped background is then eliminated using brightness and contrast enhancement. After removing the artifacts, the image is binarized using the Otsu thresholding algorithm, which highlights the lung region of the image. As some artifacts still remain, the largest contour detection algorithm is applied, and the output images are inverted. Utilizing the flood fill operation, the pixel value of the entire image except the two lung regions is set to 0 (black). After that, to get a binary mask containing only two lung-shaped white contours, the inner areas of the lung are filled with white, using the reconstruction function of skimage. Thus, a targeted binary mask is achieved. The lung portion of the image is extracted from the Raw CT scan, utilizing the Bitwise_AND function of OpenCV with the mask. Figure 9 illustrates the entire process along with the output.

##### Total Variation Denoising Method

**Figure 10:**
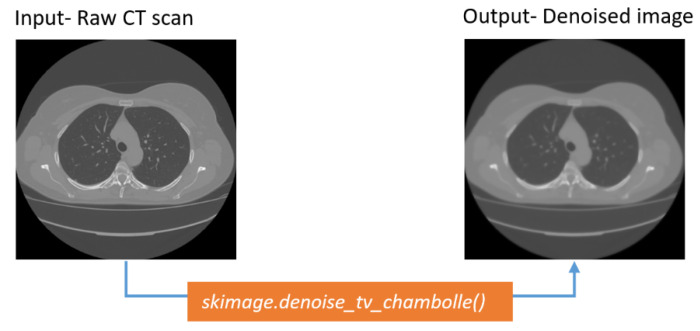
Output of TV denoising algorithm.

The key purpose of the total variation denoising method is to reduce the total variation of the pixels of a targeted image while not blurring the edges [41]. It is a noise removal process that works best with images that have a large variation in details. This technique outperforms traditional noise removal methods, such as instance linear smoothing or median filtering, which decrease noise but simultaneously smooth away relevant edges. The total variation denoising algorithm is very effective in terms of preserving edges while smoothing out the tiny noises. The outcome of the method is depicted in Figure 10.

Figure 10 shows that the output image has been denoised without losing significant information.

##### Dynamic Brightness and Contrast Adjustment

After denoising the images, the circular-shaped background is minimized by adjusting brightness and contrast, employing a dynamic threshold-based approach based on the intensity level. Brightness refers to the total lightness or darkness of an image. Contrast represents the intensity difference between the ROI and the background pixels of an image. The equation of altering brightness is:

(1) g(x)=s(x)+β

In this equation, *s*(*x*) is the input picture and *g*(*x*) is the output, after increasing or reducing the brightness by changing the value of *β*. This value will add to or deduct from every pixel a constant level of brightness.

To adjust the contrast of an image, the difference in brightness level is increased by a multiple:

(2) g(x)=a×s(x)

Here, *s*(*x*) is the source picture and *g*(*x*) is the resultant pixel value when changing contrast by adjusting the parameter value of *a*.

**Figure 11:**
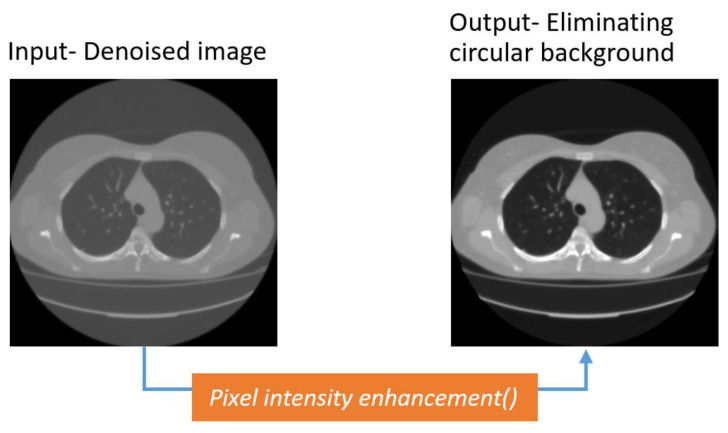
Enhanced CT scan with defocused circular background.

In most cases, not all images in a dataset have similar intensity levels. Therefore, a constant value for adjusting brightness and contrast might not work optimally for all the images and might not remove the artifacts successfully. For this reason, the mean pixel intensity of each CT scan is calculated. Depending on the value, alpha and beta are allocated so that the intensity level of all the images is adjusted and the circular-shaped background can be eliminated without losing significant details. After experimentation, a total of seven brightness contrast enhancement thresholds are derived from the mean pixel intensity values. After applying the enhancement, corresponding to the respective mean value range of that image, the circular background of the images faded (Figure 11), and further segmentation becomes easier.

It can be observed from Figure 11 that the intensity of the circular shaped background surrounding the lungs is diminished in the output image. The custom algorithm adjusts the brightness and contrasts, resulting in a defined pixel intensity in the output image.

##### Otsu Threshold Algorithm

The Otsu thresholding algorithm is widely used to convert an image from grayscale into a binary format, employing a non-linear operation [42]. The algorithm changes an image to binary format according to the intensity level of the input image based on the following conditions:

If intensity [pixel] > particular threshold, the resultant pixel = 1 (white)

Else if intensity [pixel] <= particular threshold, the equivalent output pixel = 0 (black)

Following this technique, the method finds an ideal threshold value by lessening or maximizing the within-class difference (*σ*^2^) of the foreground and background pixels. The within-class variance equations are:

(3) $$\sigma^{2}(t)=w_{0}(t)w_{1}(t){\left[\mu_{1}(t)-\mu_{2}(t)\right]}^{2}$$

(4) $$\sigma^{2}(t)=w_{0}(t)[-w_{0}(t)[\frac{\mu -\mu_{1}(t)}{1-w_{0}(t)}-\frac{\mu (t)}{w_{0}(t)}]]$$

(5) $$w_{0}(t)=\sum_{N}^{n}w_{1}(t)=1-w_{0}(t),\mu (t)=\sum t\frac{n}{N}$$

where *σ*^2^ (*t*) refers to the within-class variance, *w* indicates the weight, *μ* is the mutual mean value, and *t* refers to the threshold value. Here, $w_{0}$, $\mu_{1}$ denote the weight and average value of the background pixels, respectively, and $w_{1}$, $\mu_{2}$ correspondingly denote the weight and average value of the foreground pixels.

**Figure 12:**
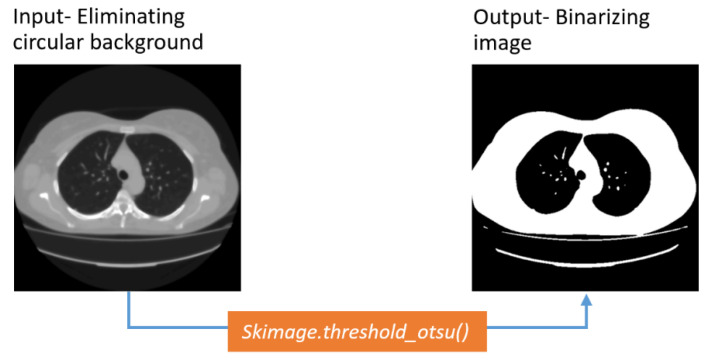
Output of Otsu thresholding.

The aim of applying Otsu thresholding, to make the image binary, has been successfully achieved, as can be seen in Figure 12.

##### Largest Contour Detection

As can be observed from Fig 3-D, thin curved lines are seen at the bottom of the lung portions. To remove the curved lines along with other artifacts (if they exist) the largest contour detection approach is adopted. In this method, the biggest contour in the image, the two lungs and the surrounding white region, is extracted. This contour is identified utilizing the findContours () and max () functions of OpenCV. The findContours () function takes the binarized CT scan of the previous step as input, calculates all possible contours present in the image, and stores the coordinates of the contours in memory. The largest contour in terms of area is then identified with the max () function. The RETR_EXTERNAL argument of the find Contour () function is able to retrieve the largest contour’s coordinates and, with the CHAIN_APPROX_SIMPLE argument, the edge coordinates of the largest object are retained. Finally, with the drawContours () function, an outline of this largest object is found utilizing the edge information provided by findContours () function. After completion of the process, a binary mask containing only the largest blob is achieved.

**Figure 13:**
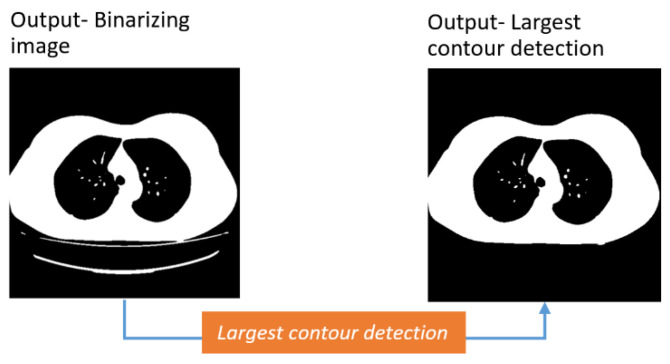
Curved line removal with largest contour detection.

After detecting the largest contour, the curved lines, along with other artifacts, have been removed, as can be seen from the output image in Figure 13.

##### Inverting the Image

After removing the artifacts successfully, the images are inverted. In this way, the white pixels become black, and all the black pixels become white.

**Figure 14:**
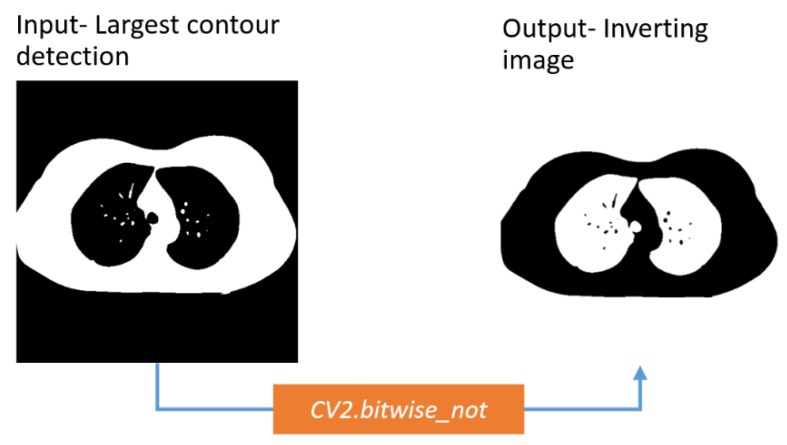
Output after inverting image.

The background and the lungs are turned white (Figure 14).

##### Flood Fill Operation

**Figure 15:**
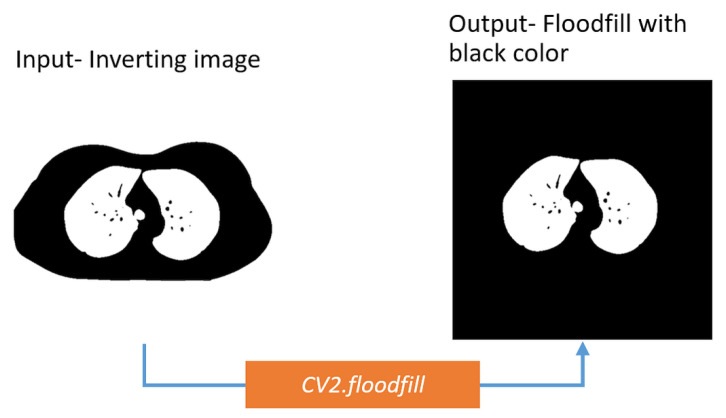
Making a binary mask containing the lung area using floodfill algorithm.

After inverting the images, it is found that there is a circular-shaped border surrounding the lungs. This can be removed using the flood fill operation. In this algorithm, the starting point is specified, and the color of neighboring pixels of a particular boundary is changed from that point. The algorithm uses the following equation:

(6) $$X_{k}=(X_{k-1}⊗B)\cap A^{c}$$

where *B* refers to the structuring element, *k* is the pixel index of the input picture which will be transformed or filled, and *k* − 1 specifies the adjacent pixels. *A* represents the set comprehending a subset. This algorithm is applied to the four corners of the image. Suppose the images are of ‘h’ height and ‘w’ width. Thus, the coordinates of the four corners are the upper left corner (0, 0), upper right corner (0, w), downward left corner (h, 0), and downward right corner (h, w). The flood fill algorithm is applied from these four corners coordinates of the images. After applying the algorithm, the surrounding circular-shaped object is filled with a pixel value of 0, and the lung regions are white. Figure 15 shows that, due to flood filling, only the lung portions remain white.

##### Inner Hole Filling

The lungs contain bronchi, arteries, and other vessels. To achieve a clean binary mask, these should be removed. Therefore, the flood fill operation is again used to fill the inner holes of the lungs, using a pixel value of 1.

**Figure 16:**
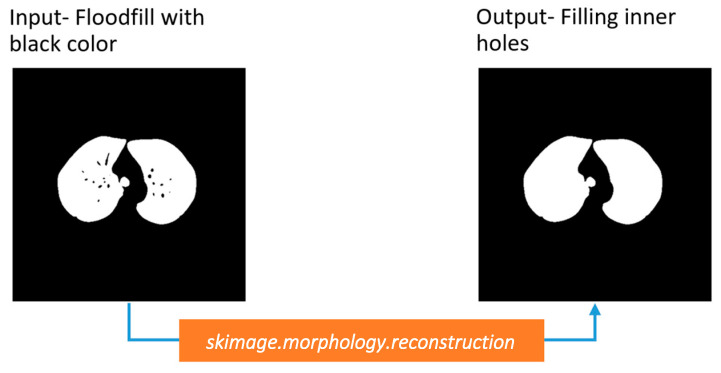
Filling inner holes of the binary mask.

Finally, a binary mask of just the two lungs is obtained, as shown in Figure 16.

##### Extracting Lung

**Figure 17:**
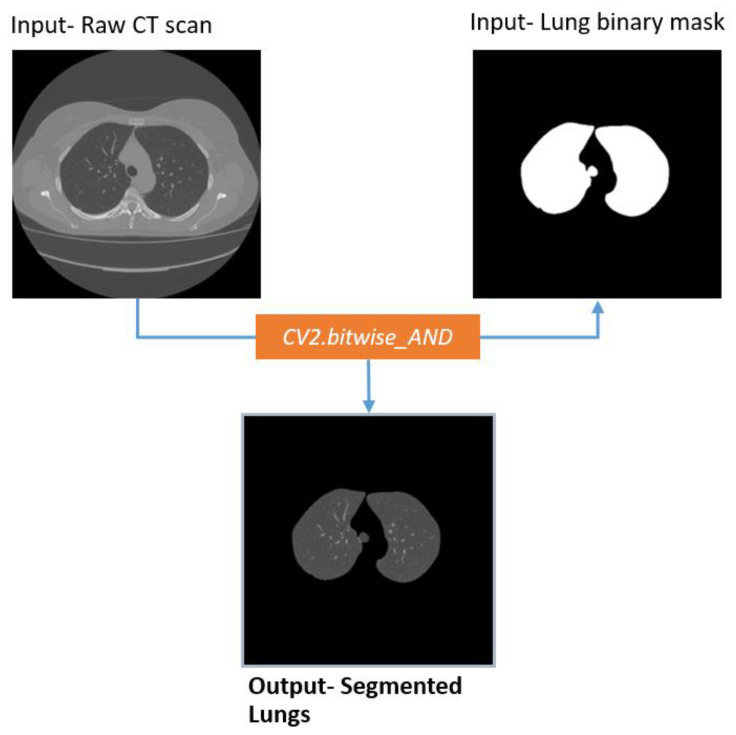
Extracted lungs from Raw CT scan using lung binary mask.

The segmented binary mask is fused with the source image employing a bitwise_AND operation (Figure 17). Thus, the lungs are segmented without losing any details.

Figure 17 illustrates that, after merging the binary mask with the raw CT image, the output contains only the lungs.

**Figure 18:**
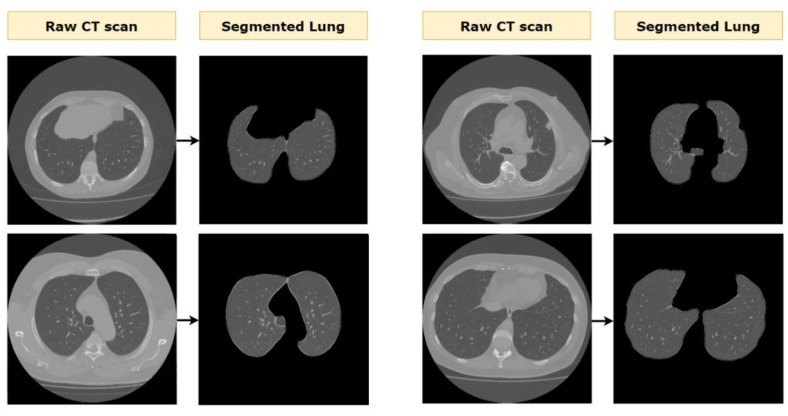
Example outcomes of the lung segmentation process.

Examples of lung segmentation on various CT scans are presented in Figure 18.

#### 2.5.2. Detection of BA from the Lungs

To highlight BA pairs from the segmented lungs, four approaches are adopted: (a) the Hessian-based method, (b) the region-growing algorithm, (c) the clustering-based method, and (d) the color-coding-based method. The motivation for considering four approaches is to compare their effectiveness in terms of disease classification performance using deep learning techniques.

**Figure 19:**
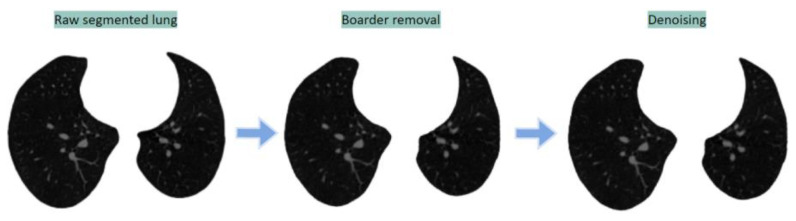
Preprocessing of segmented lung images.

The segmented images are first preprocessed by eroding the adjacent thin border surrounding the lungs and eliminating tiny noises within the lungs with morphological erosion and median filtering, respectively. This helps to distinguish the arteries more clearly from the dark regions of the lungs. Figure 19 shows the output images after applying these two methods sequentially.

##### Hessian-Based Method

**Figure 20:**
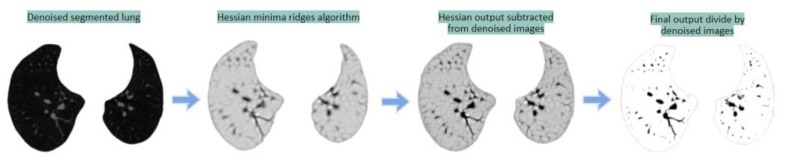
Hessian-based segmentation process.

The Hessian matrix-based ridge detection algorithm is applied to the preprocessed images, delivering local minimum ridges information [43]. The arteries appear darker, and everything else is lightened. This is a multiscale segmentation method that utilizes the central medialness adaptive principle. Depending on the eigenvalues of the Hessian matrix of the image intensities, the ridges of the image are highlighted. Figure 20 shows the output of the Hessian-based approach after experimenting with different sigma values. A sigma value of 2.5 gave the best results.

The Hessian minima ridges algorithm is applied to the denoised image, enhancing the vessels. The output from the Hessian algorithm is then subtracted from the denoised image to boost the intensity level of the arteries in a light background. Finally, the subtracted image is divided by the denoised image resulting in a grayscale image with improved intensity levels.

##### Region-Growing Method

**Figure 21:**
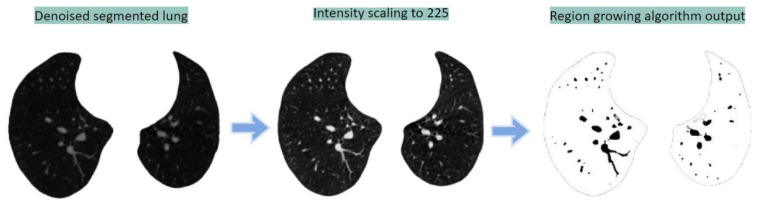
Region-growing-based segmentation process.

The region-growing algorithm is explored as the second BA detection approach in this study. This method is a region or pixel-based segmentation approach which works by selecting initial seed points. The algorithm studies adjacent pixels of the primary seed points in an iterative manner to determine whether the neighboring pixel can be added to the area based on intensity similarity [44]. The process follows an iterative method, accumulating pixels into greater regions depending on pre-initialized seed pixels and growing conditions. The middle pixel of both lungs is calculated and fed to the region-growing algorithm as the start pixel. From these seed points and according to predefined threshold conditions, the regions start growing. Figure 21 represents the output image after the completion of the process.

The intensity level of the denoised images is scaled so that the lowest value is 0 and highest intensity value is 255. In this way the arteries become more visible. The region-growing algorithm is applied to the enhanced image, see Figure 21. After experimentation, the intensity thresholds for region-growing algorithm are set to 220–255.

**Figure 22:**
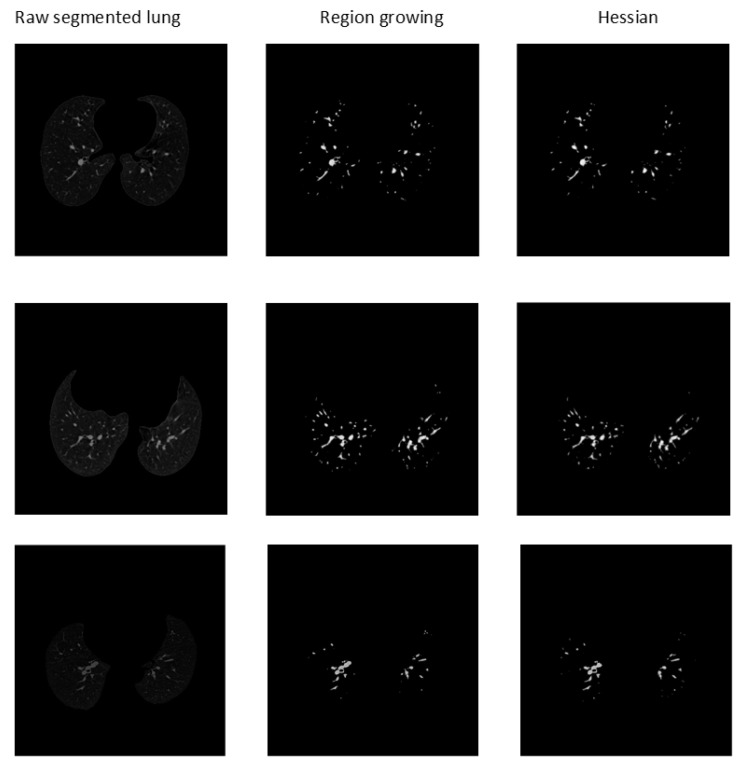
Segmented arteries from CT scans using region-growing and Hessian methods.

Examples of the outcomes utilizing the masks generated from the Hessian and region-growing-based methods are presented in Figure 22.

##### Clustering Approach

**Figure 23:**
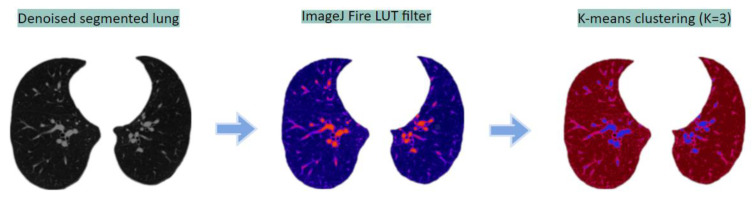
Segmentation of BA from the lung portions using clustering approach.

In this method, the raw segmented image is first color-coded using ImageJ [45] software’s ‘Fire filter, which enhances the BA pairs. Afterward, a K-means clustering algorithm [46] is applied to the color-coded image with a K value of 3. This is an unsupervised algorithm that is used to segment the ROI from the background K-centroids. Figure 23 depicts the output of the approach.

**Figure 24:**
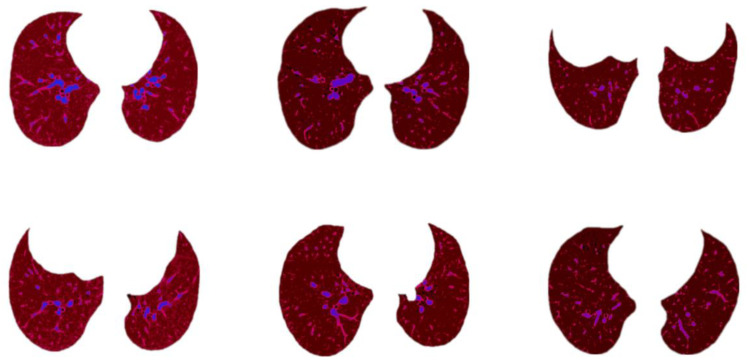
Sample outputs of clustering approach.

Some outputs achieved from this approach are shown in Figure 24.

##### Color-Coding Approach

**Figure 25:**
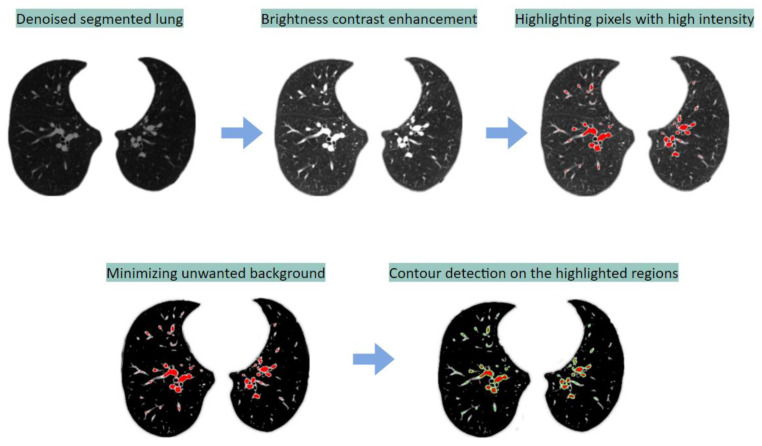
Segmentation of BA from the lungs using the color-coding method.

First, the segmented lung image is enhanced using alpha-beta correction. Employing a dynamic thresholding algorithm, the bright pixels, which can be considered as potential arteries, are assigned a red color pixel value. Then, again using dynamic thresholding, the pixels less than the mean pixel values are made 0. Finally, all the red objects are detected and marked with green lines using contour detection and contour drawing methods. Figure 25 depicts the process.

**Figure 26:**
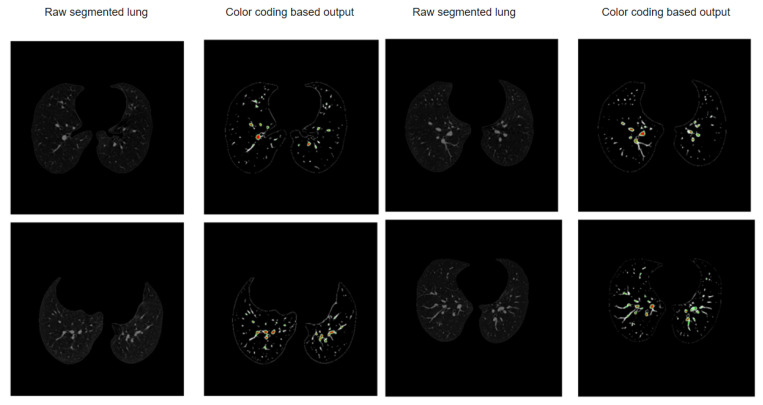
Sample outputs obtained with the color-coding-based approach.

Figure 26 shows some sample outputs obtained with this approach.

### 2.6. Classification Using Deep Learning

Four BA datasets are obtained from the four approaches. Two deep learning networks, (a) a CNN and (b) a CNN with LSTM combined with an attention mechanism, are applied to classify the images of these datasets into three classes. In order to develop a model with the best possible performance, an ablation study using the segmented raw lung dataset is conducted on the CNN architecture to optimize the hyper-parameters. LSTM and attention mechanism layers are added to the CNN architecture achieved after conducting the ablation study.

#### 2.6.1. CNN-Based Classification

**Figure 27:**
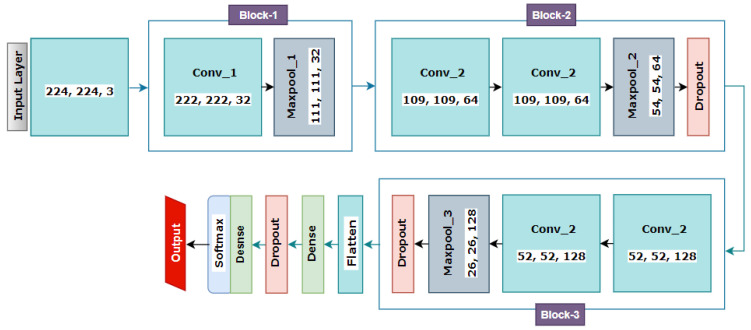
Proposed CNN model.

As deep learning models require extensive computational resources and training time, we try to keep the architecture of the model as shallow as possible to minimize time and computational complexity [47]. The proposed CNN architecture has different components and layers, such as the input layer, convolutional, pooling, fully connected, dense, dropout, and a final output dense layer. Figure 27 illustrates the architecture of the network after the ablation study.

The proposed network contains 14 layers containing five convolutional layers, three max-pooling, three dropout layers having factor 0.5, one dense layer having factor 128, and a final dense layer for classification. A Flatten layer is added prior to the final classification dense layer. All the convolution layers have the same 3 × 3 kernel size, and the PReLU activation function is added to introduce non-linearity. The kernel size for the max-pooling layer is set to 2 × 2. As can be seen from Figure 27, the architecture has three blocks. In block-1, the input layer is connected to the first Conv2D layer, which contains 32 kernels and 788,992 trainable parameters, and the dimensions of the input image size are 224 × 224 × 3. The first Conv2D layer is followed by a maxpool layer, scaling down the feature maps attained from the first Conv2D layer into half of its preceding size (from 222 × 222 to 111 × 111). Block-2 and -3 have the same configuration, except for the number of kernels in the Conv2D layers. In each block, two Conv2D layers having the same configuration are added. The kernel number is 64 for the Conv2D layers of the second block and 128 for the Conv2D layers of the third block. These two convolution layers are again followed by a max-pool layer which reduces the size of the resultant feature maps from 107 × 107 to 53 × 53 for the first block and from 49 × 49 to 24 × 24 for the second block. A dropout layer with a factor of 0.5 is added after the max-pooling for both blocks. Block-2 and block-3 represent 384,832 and 95,776 trainable parameters, respectively. A Flatten layer outputs the feature maps generated from block-3 into a 1D vector. A dense layer, comprising 128 neurons equipped with PReLU activation function, is added after the Flatten layer. Another dropout layer with a value of 0.5 is attached afterward. As the dataset has three classes, the classification dense layer, which is also known as the fully connected layer (FC), contains three neurons and is equipped with the Softmax activation function.

#### 2.6.2. CNN + LSTM + Attention Mechanism-Based Classification

**Figure 28:**
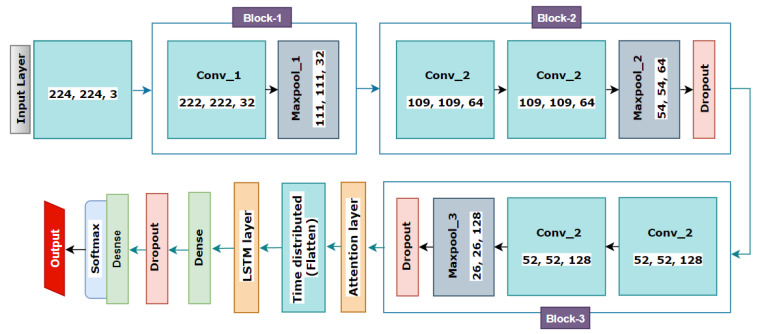
Proposed CNN model modified with attention mechanism and LSTM.

To further improve the base CNN model, experimentation with LSTM [48,49] and attention mechanism [50] is carried out. The attention mechanism is an innovative method to apprehend long-range feature interactions, resulting in improved interpretation capability of CNNs. This mechanism concentrates the decoder’s attention on the most significant features, employing a weighted sum of all preceding hidden states [51]. The internal memory of the LSTM layer makes it possible to learn from experiences regarding long-term states [52]. Here, the nodes between the layers from a directed graph are associated with a chronological order that is considered an input. CNN combined with the LSTM layer may improve the classification performance significantly [49]. Therefore, in the second proposed model, two additional layers, LSTM, and attention mechanism, have been added to the previously described CNN network. Figure 28 illustrates the network architecture of the CNN with LSTM and attention mechanism-based model.

To integrate this functionality into our CNN model, an attention layer is added between the third block and the Flatten layer. In addition, an LSTM layer is added after the Flatten layer. While implementing LSTM, the Flatten layer is modified with a time-distributed function to make the flattened output compatible with the newly added LSTM layer. The LSTM layer is equipped with 256 neurons and an additional dropout of 0.5 to prevent over-fitting of the model. The output of the LSTM layer is fed to the previously existing dense layer. Every other aspect of the CNN model remains unchanged.

#### 2.6.3. Dataset Split and Training Strategy

Datasets acquired from the four approaches are split into training, validation, and testing datasets utilizing a 70:20:10 ratio [53]. ‘Categorical cross-entropy’ is employed as the loss function of the proposed model. The formula for the categorical cross-entropy loss function is as follows [54]:

(7) $$\mathrm{Loss}(d,v)=-\sum_{j=0}^{m}\sum_{i=0}^{n}(d_{ij}*\log({\hat{v}}_{ij}))$$

where true label and predicted labels are represented by *d* and *v*, respectively. In addition, *m* denotes the batch size, and *n* denotes the total number of classes. The probability predicted by the model is ${\hat{v}}_{ij}$ for the *i*th observation of the *j*th category. The Nadam optimizer with a learning rate of 0.0008 and a batch size of 64 is utilized [55]. Two computers were used for this research. They are equipped with NVidia GeForce GTX 1660 GPU, Intel Core i5-8400 Processor, 256 GB DDR4 SSD for storage, and 16 GB of Memory.

## 3. Results

In this section, the performance of our four approaches is analyzed using several evaluation metrics. Results of both deep learning models for the four approaches are discussed.

### 3.1. Developing the CNN Model Employing Ablation Study

A total of six ablation case studies are carried out using the raw segmented dataset to acquire the optimal configuration of the model by altering different hyper-parameters. The results are shown in Table 2.

Ablation Study 1: Kernel size.

Various convolutional layer kernel sizes have been investigated in this study. Four kernel sizes (2, 3, 4, and 5) are trialed. A kernel of size 3 obtained the maximum accuracy, of 83.68%, with a low per epoch training time of 135 s (Table 2). So, a kernel size of 3 is used in the CNN model.

Ablation Study 2: Loss function

To achieve peak results, various loss functions are evaluated: categorical cross-entropy, mean absolute error, and mean squared error. The model achieved the highest test accuracy of 83.68% (Table 2) with the categorical cross-entropy loss function. Therefore, this loss function is utilized in the final model.

Ablation Study 3: Pooling layer

Experimentation with both maxpooling and average pooling layers demonstrates that the highest performance is achieved with the maxpooling layer resulting in an accuracy of 83.83%. Hence maxpooling layers are utilized in the final CNN model.

Ablation Study 4: Activation function

Various activation functions, Exponential Linear Units (ELU), Tanh, ReLU, SoftPlus, and SoftSign, are tested with the CNN model in order to find the best suited for our model (Table 2). Results show that PReLU performed the best, with an accuracy of 84.52%. PReLU is selected for further ablation studies.

Ablation Study 5: Optimizer

Five distinct optimizers are used in the experiments: Adam, Nadam, SGD, Adamax, and RMSprop. Learning rates for all the optimizers are set to 0.001. The highest test accuracy, 85.28%, was achieved with the Nadam optimizer (Table 2). The Nadam optimizer is chosen for the final CNN model.

Ablation Study 6: Learning rate

Table 2 displays the results of evaluating the Nadam optimizer at various learning rates: 0.01, 0.008, 0.001, and 0.0008. The best results are obtained with a learning rate of 0.0006, which results in a test accuracy of 85.69% while maintaining a per-epoch training duration of 135 s. Thus, the Nadam optimizer with a learning rate of 0.0008 is selected.

**Figure 29:**
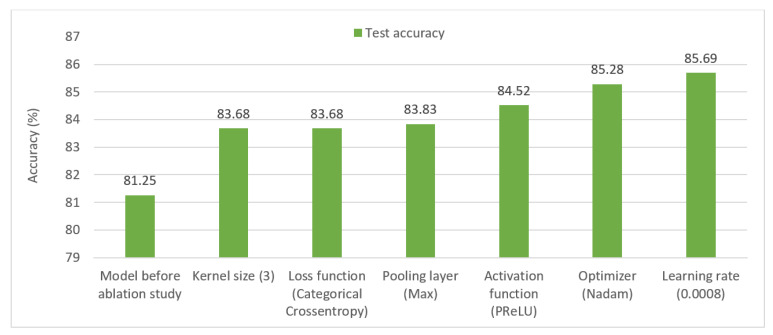
Gradual increase of test accuracy in various ablation studies.

The gradual increase oin accuracy through ablation case studies is shown in Figure 29.

### 3.2. Classification Performance of CNN Model

The developed CNN network is trained using the four datasets generated with the Hessian, region-growing, color-coding, and clustering methods. The training accuracy, validation accuracy, test accuracy, recall, specificity, precision, and F1 score [56] are derived for every individual dataset. Table 3 depicts the classification results acquired from the four datasets for the CNN model.

It can be observed from Table 3 that in all cases the model yields satisfactory training accuracies of 94.65%, 92.57%, 95.41%, and 97.76% for the Hessian, region-growing algorithm, color-coding, and clustering-based segmented datasets, respectively. However, for the Hessian and region-growing algorithm-based datasets, the validation and test accuracies drop significantly, to 88.52% and 88.78%, respectively, for the Hessian dataset and to 86.82% and 87.14%, respectively, for the region-growing algorithm. This is a clear identification of overfitting. For other metrics, the results were below 90% as well, except for specificity. A specificity of 93.37% and 91.31% is achieved for the Hessian and region-growing algorithm, respectively. In contrast, the color-coding and clustering-based approaches provide promising outcomes, yielding validation accuracies of 94.85% and 96.12% and test accuracies of 92.36% and 95.84%, respectively. Looking at the other metrics, it is found that all the values are satisfactory for these approaches (>90%), though the clustering-based approach outperforms the others with a recall of 97.15%, a precision of 94.79%, a specificity of 98.88%, and an F1 score of 96.06%. However, for the color-coding-based approach, a satisfactory recall of 94.72%, a specificity of 97.18%, a precision of 90.66%, and an F1 score of 92.75% were also achieved.

We have also derived test accuracies for all four approaches for individual classes. Table 4 lists the classification results for individual classes of the four datasets.

It can be seen that the best performance is achieved with the color-coding and clustering-based approaches for distinct classes as well. No significantly lower accuracy is observed for any individual class, which validates the robustness of these two approaches. For the clustering-based method, test accuracies of 94.61%, 95.34%, and 96.38% are achieved for Non-COVID, COVID, and CAP, respectively. Likewise, for the color-coding approach, test accuracies of 91.86%, 92.14, and 93.27% are achieved for Non-COVID, COVID, and CAP, respectively. The Hessian and region-growing algorithms resulted in comparatively poor outcomes for all classes, yielding test accuracies below 90%.

### 3.3. Classification Performance of CNN with LSTM and Attention Mechanism Model

The proposed CNN with LSTM and Attention mechanism model is trained with four datasets, produced with the Hessian, region-growing, color-coding, and clustering-based approaches. For each of the approaches, the training accuracy, validation accuracy, test accuracy, recall, specificity, precision, and F1 scores are calculated. Table 4 shows the classification results acquired with the four datasets for the CNN with LSTM and attention mechanism model.

From Table 5 it can be seen that, like the CNN model, occurrence of over-fitting is observed for both the Hessian and region growing algorithms, yielding training accuracies 95.90% and 93.21% and validation accuracies 88.95% and 87.58%, respectively. However, with this model, the test accuracy increases slightly, by approximately 1%, for both methods. For color-coding and clustering, the test accuracy improves by around 2% in both cases. With the clustering approach, a training accuracy of 98.68%, validation accuracy of 96.83%, test accuracy of 97.12%, recall of 98.26%, specificity of 99.02%, precision of 99.02%, and F1 score of 97.57% are achieved. Though the clustering-based approach outperforms the other three techniques, a promising performance is also acquired with the color-coding-based approach. In this case, a training accuracy of 97.52%, validation accuracy of 96.47%, test accuracy of 94.61%, recall of 96.44%, precision of 92.22%, specificity of 98.73%, and an F1 score of 94.97% are obtained.

In addition, the test accuracy for individual classes was derived for all four approaches. Table 6 lists the test accuracies acquired for individual classes of the four datasets.

Table 6 shows that for distinct classes, the best performance is attained with the color-coding and clustering-based approaches. The model shows no bias to any of the classes, validating the robustness of our proposed network with these two approaches. In the color-coding-based method, for Non-COVID, COVID, and CAP, test accuracies of 93.86%, 94.62%, and 95.71% are acquired, respectively. Likewise, for the clustering approach, for Non-COVID, COVID, and CAP, test accuracies of 96.61%, 97.09%, and 99.23% are achieved. In contrast, both the Hessian and region-growing algorithms yielded relatively poor results for all classes, with test accuracies below 90%.

**Figure 30:**
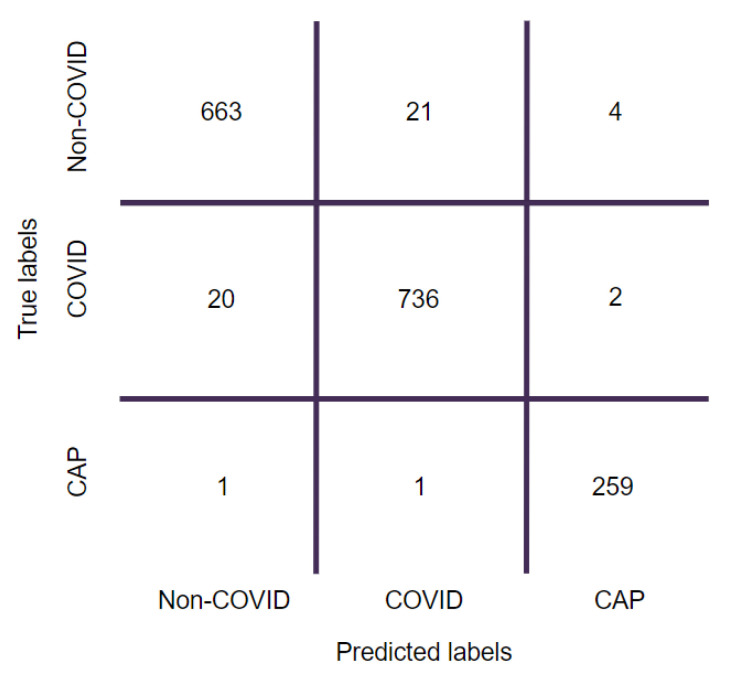
Confusion matrix generated for the CNN with LSTM and attention mechanism-based model for the clustering-based approach.

**Figure 31:**
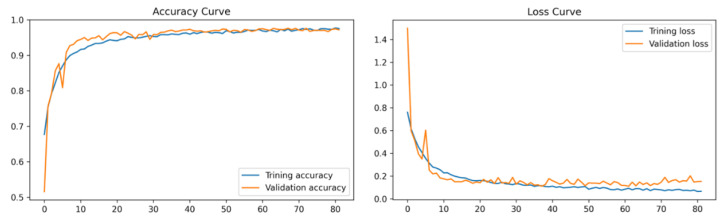
Accuracy and loss curves for CNN with LSTM and attention mechanism model trained on the clustering-based approach dataset.

For the highest performing clustering-based approach trained on the CNN with LSTM and attention mechanism model, a confusion matrix and training curves were derived. They are presented in Figure 30 and Figure 31, respectively.

In the confusion matrix (Figure 30), true labels of the images are indicated by the row values, and the values predicted by the model are given by the column values. Diagonal values indicate correct predictions. The highest proportion of correct predictions is observed for CAP, with only two misclassifications. The model performed quite well in predicting the other two classes as well, with misclassifications below 25. This shows that the network is not biased to any specific class and yields good classification accuracies across all classes.

Based on the accuracy curves (Figure 31), there is no evidence of overfitting while training the model, as both the training and validation curves are seen to be smoothly converging, having a minimal gap between them. Moreover, the loss curves are steadily decreasing from the first to the last epoch, with a small gap between them. No indication of overfitting and underfitting is noticed during the training phase of the model.

### 3.4. Performance Comparison of the CNN + LSTM + Attention Mechanism Model with the Segmented CT Scan and Highlighted CT Scan

To assess the significance of considering BA alterations in COVID-19 and pneumonia diagnosis, a performance comparison is presented based on test accuracies for the four approaches. Table 7 shows the results.

It can be observed from Table 7 that both the color-coding and the clustering-based approaches had better results than the proposed model with segmented lung images. In terms of test accuracies, a 5–8 % increase is observed, along with a 5–8% boost in F1 score for the color-coding and clustering-based approaches compared to the segmented lung CT scans. This is the consequence of enhancing the CT scans, which visually emphasizes characteristics of the CT scans which help to distinguish the different classes. The performance boost in the color-coding and clustering-based approaches justify the approach taken in this research. The CNN with LSTM and attention mechanism model is a robust classification model.

### 3.5. Stability Analysis of the Proposed CNN + LSTM + Attention Mechanism Model in Terms of Complexity

CNN with LSTM and attention mechanism model has proven to be quite effective in accurately classifying CT scans into three classes with a high accuracy of 97.12% on a clustering-based dataset and 94.61% on a color-coding-based dataset (Table 7). The model is trained multiple times with clustering-based and color-coding-based datasets utilizing various K-fold configurations to measure the performance consistency of the model. A total of 8 k fold configurations are experimented with where the K value ranges from 3 to 20 (Table 8). The proposed model is also evaluated in terms of complexity and training time for the two best datasets (color coding and clustering).

It can be observed from Table 8 that the model yields accuracies close to the highest accuracy in every fold for both the color-coding and clustering datasets while maintaining identical training periods. The results of K-fold cross-validations indicate that consistent performance is achieved across all configurations. The accuracy is in the range of 96.95–97.20% for the clustering-based dataset and in the range of 94.40–94.65% for the color-coding-based dataset. Furthermore, a stable per epoch training time of 130–135 s and total training time of 3.5–3.75 s is recorded across all k-fold configurations for both datasets. A similar RAM usage of 61–63% is recorded for both datasets over all k-folds, where the total usable RAM is 12 GB. This demonstrates the stability of the model in terms of complexity while trained with two different preprocessed datasets. Furthermore, no significant drop in performance could be observed for any k-fold configuration, which further validates the consistency of the performance.

### 3.6. Discussion

Based on previous literature, features such as bronchi, arteries, and blood vessels can have an impact in distinguishing lung disease classes from CT scans. In recent times, COVID-19 and pneumonia have been getting researchers’ attention. Several state-of-the-art automated methods have been developed for classifying diseases. BA distortions are also considered important indicators of lung disease severity. In COVID-19 research, this should be taken into account. In this study, classification is performed using different methods to highlight BA pairs. Based on the high classification accuracies obtained with the color-coding and clustering-based approaches, it can be stated that the BA pairs were successfully highlighted. For the other two approaches, the Hessian and the region-growing algorithms, it could be observed that several important regions, especially bronchi, were eliminated. This may be the cause of the comparatively poor classification outcomes. The possible reason for the high classification accuracy while applying color-coding and clustering-based methods can be that no relevant information about the lungs is eliminated. The BA pairs are highlighted while other information is preserved. In clinical appliances, the experts look for different alterations of the lung information to identify the disorder. Our approach may assist clinicians in diagnosing lung disorders more accurately as no relevant information is lost from the image, but the important features are highlighted by the proposed approach.

## 4. Conclusions

In this research, a robust automated framework is introduced, combined with the segmentation of lungs, detection of BA changes, and categorization of lung disorders introducing deep learning techniques. To detect the BA structural distortions, the lungs are first segmented from the CT scans utilizing several image preprocessing algorithms. We applied four automated approaches to the segmented dataset to detect BA structural alterations associated with lung disorders. Two different deep learning architectures: (a) a CNN and (b) a CNN with LSTM and an attention mechanism, are employed to classify lung diseases using the datasets acquired from the four approaches, and the performance is compared. CNN with LSTM and an attention mechanism outperforms CNN, yielding a test accuracy of 97.12% when applied to the dataset achieved from the clustering-based detection method. CNN achieved a test accuracy of 95.84%. Similarly, for the other approaches, a 1–2% accuracy gain is observed with the improved model. The accuracy and loss curves and the outcomes achieved from the k-fold cross-validation confirm the robustness of the approach with no indication of overfitting.

## Figures and Tables

**Table 1:** Description of dataset.

Name	Description
Total amount of CT scans	17,104
Dimension	512 × 512
Images type	CT scan
Colour Grading	Gray scale
Non-COVID	6983
COVID	7593
CAP	2618

**Table 2:** Result of ablation studies.

Ablation Study 1: Changing Kernel Size				
Configuration	Kernel Size	Epoch × Training Time	Test Accuracy	Finding
1	4	100 × 161 s	81.25%	Previous accuracy
2	3	100 × 135 s	83.68%	Highest accuracy
3	2	100 × 135 s	78.54%	Accuracy dropped
4	5	100 × 170 s	80.71%	Accuracy dropped
Ablation Study 2: Changing the Loss Function				
Configuration	Loss Function	Epoch × Training Time	Test Accuracy	Finding
1	Categorical Cross-entropy	100 × 135 s	83.68%	Highest accuracy
2	Mean Squared Error	100 × 135 s	78.15%	Accuracy dropped
3	Mean absolute error	100 × 135 s	79.55%	Accuracy dropped
Ablation Study 3: Changing the Type of Pooling Layer				
Configuration	Type of Pooling Layer	Epochs × Training Time	Test Accuracy	Findings
1	Max	100 × 135 s	83.83%	Highest accuracy
2	Average	100 × 135 s	83.68%	Previous accuracy
Ablation Study 4: Changing the Activation Function				
Configuration	Activation Function	Epochs × Training Time	Test Accuracy	Findings
1	Tanh	100 × 135 s	79.74%	Accuracy dropped
2	ReLU	100 × 135 s	83.83%	Previous accuracy
3	PReLU	100 × 135 s	84.52%	Highest accuracy
4	Leaky ReLU	100 × 135 s	83.33%	Improved accuracy
Ablation Study 5: Changing Optimizer				
Configuration	Optimizer	Epochs × Training Time	Test Accuracy	Findings
1	Adam	100 × 135 s	84.52%	Previous accuracy
2	Nadam	100 × 135 s	85.28%	Highest dropped
3	SGD	100 × 135 s	79.28%	Accuracy dropped
4	Adamax	100 × 135 s	84.27%	Accuracy dropped
5	RMSprop	100 × 135 s	83.95%	Accuracy dropped
Ablation Study 6: Learning Rate				
Configuration	Learning Rate	Epochs × Training Time	Test Accuracy	Findings
1	0.0001	100 × 135 s	85.42%	Improved accuracy
2	0.001	100 × 135 s	85.28%	Previous accuracy
3	0.008	100 × 135 s	84.85%	Accuracy dropped
4	0.0008	100 × 135 s	85.69%	Highest accuracy

**Table 3:** Evaluation metrics of all methods with CNN.

Measure	Hessian	Region Growing	Color-Coding	Clustering
Training accuracy	94.65%	92.57%	95.41%	97.76%
Validation accuracy	88.52%	86.82%	94.85%	96.12%
Test accuracy	88.78%	87.14%	92.36%	95.84%
Recall	89.68%	89.87%	94.72%	97.15%
Specificity	93.37%	91.31%	97.18%	98.88%
Precision	87.79%	86.79%	90.66%	94.79%
F1 score	88.71%	86.83%	92.75%	96.06%

**Table 4:** Class-based accuracy of all methods with CNN.

Class	Hessian	Region Growing	Color-Coding	Clustering
Non-COVID	85.37%	86.87%	91.86%	94.61%
COVID	87.61%	86.15%	92.14%	95.34%
CAP	89.38%	88.87%	93.27%	96.38%
Overall accuracy	88.78%	87.14%	92.36%	95.84%

**Table 5:** Evaluation metrics of all methods with CNN modified with attention mechanism and LSTM.

Measure	Hessian	Region Growing	Color-Coding	Clustering
Training accuracy	95.90%	93.21%	97.52%	98.68%
Validation accuracy	88.95%	87.58%	96.47%	96.83%
Test accuracy	89.61%	88.28%	94.61%	97.12%
Recall	91.46%	90.32%	96.44%	98.26%
Specificity	94.88%	92.68%	98.73%	99.02%
Precision	88.92%	87.85%	92.22%	99.02%
F1 score (F1)	89.58%	88.43%	94.97%	97.57%

**Table 6:** Per-class accuracy of all methods with CNN modified with attention mechanism and LSTM.

Class	Hessian	Region Growing	Color-Coding	Clustering
Non-COVID	88.67%	87.43%	93.86%	96.61%
COVID	88.24%	86.54%	94.62%	97.09 %
CAP	90.27%	89.51%	95.71%	99.23%
Over all accuracy	89.56%	88.28%	94.61%	97.12%

**Table 7:** Performance of proposed model on segmented CT scan dataset, color-coding-based approach, and clustering-based approach.

Measure	Segmented Lung CT Scan	Color-Coding	Clustering
Training accuracy	92.45%	97.52%	98.68%
Validation accuracy	89.39%	96.47%	96.83%
Test accuracy	87.69%	94.61%	97.12%
Recall	89.44%	96.44%	98.26%
Specificity	93.73%	98.73%	99.02%
Precision	83.09%	92.22%	99.02%
F1 score (F1)	87.83%	94.97%	97.57%

**Table 8:** Stability of the proposed model in terms of complexity.

Dataset	K-Fold Configurations	Accuracy (%)	Per Epoch Training Time (Second)	Total Training Time (Hour)	RAM Usage
Color coding	3 fold	94.48	130–135	3.5–3.75	62%
	5 fold	94.55	130–135	3.5–3.75	61%
	7 fold	94.51	130–135	3.5–3.75	62%
	10 fold	94.59	130–135	3.5–3.75	63%
	13 fold	94.63	130–135	3.5–3.75	62%
	15 fold	94.65	130–135	3.5–3.75	62%
	17 fold	94.58	130–135	3.5–3.75	61%
	20 fold	94.63	130–135	3.5–3.75	63%
Clustering	3 fold	96.95	130–135	3.5–3.75	63%
	5 fold	97.04	130–135	3.5–3.75	64%
	7 fold	97.02	130–135	3.5–3.75	63%
	10 fold	97.18	130–135	3.5–3.75	65%
	13 fold	97.20	130–135	3.5–3.75	64%
	15 fold	96.98	130–135	3.5–3.75	63%
	17 fold	97.06	130–135	3.5–3.75	63%
	20 fold	97.11	130–135	3.5–3.75	65%

## Data Availability

Large COVID-19 CT Scan Slice Dataset|Kaggle Available online: https://www.kaggle.com/datasets/maedemaftouni/large-covid19-ct-slice-dataset (accessed on 26 November 2022).

## References

[1] Han B.K., Rigsby C.K., Hlavacek A., Leipsic J., Nicol E.D., Siegel M.J., Bardo D., Abbara S., Ghoshhajra B., Lesser J.R. (2015). Computed Tomography Imaging in Patients with Congenital Heart Disease Part I: Rationale and Utility. An Expert Consensus Document of the Society of Cardiovascular Computed Tomography (SCCT): Endorsed by the Society of Pediatric Radiology (SPR) and the North American Society of Cardiac Imaging (NASCI). J. Cardiovasc. Comput. Tomogr..

[2] Park H.S., Kim Y., Kim H.Y., Zo J.I., Lee J.H., Lee J.S. (2007). Bronchial Artery and Systemic Artery Embolization in the Management of Primary Lung Cancer Patients with Hemoptysis. Cardiovasc. Intervent. Radiol..

[3] Ko J.P., Naidich D.P. (2004). Computer-Aided Diagnosis and the Evaluation of Lung Disease. J. Thorac. Imaging.

[4] Kwee T.C., Kwee R.M. (2020). Chest Ct in COVID-19: What the Radiologist Needs to Know. Radiographics.

[5] Pizzutto S.J., Hare K.M., Upham J.W. (2017). Bronchiectasis in Children: Current Concepts in Immunology and Microbiology. Front. Pediatr..

[6] Wu J., Bracken J., Lam A., Francis K.L., Ramanauskas F., Chang A.B., Robinson P., McCallum P., Wurzel D.F. (2021). Refining Diagnostic Criteria for Paediatric Bronchiectasis Using Low-Dose CT Scan. Respir. Med..

[7] Kuo W., de Bruijne M., Petersen J., Nasserinejad K., Ozturk H., Chen Y., Perez-Rovira A., Tiddens H.A.W.M. (2017). Diagnosis of Bronchiectasis and Airway Wall Thickening in Children with Cystic Fibrosis: Objective Airway-Artery Quantification. Eur. Radiol..

[8] Matsuoka S., Uchiyama K., Shima H., Ueno N., Oish S., Nojiri Y. (2003). Bronchoarterial Ratio and Bronchial Wall Thickness on High-Resolution CT in Asymptomatic Subjects: Correlation with Age and Smoking. Am. J. Roentgenol..

[9] Lynch D.A., Hay T., Newell J.D., Divgi V.D., Fan L.L. (1999). Pediatric Diffuse Lung Disease: Diagnosis and Classification Using High-Resolution CT. Am. J. Roentgenol..

[10] Ambrosetti M.C., Battocchio G., Zamboni G.A., Fava C., Tacconelli E., Mansueto G. (2020). Rapid Onset of Bronchiectasis in COVID-19 Pneumonia: Two Cases Studied with CT. Radiol. Case Reports.

[11] Kapur N., Masel J.P., Watson D., Masters I.B., Chang A.B. (2011). Bronchoarterial Ratio on High-Resolution CT Scan of the Chest in Children without Pulmonary Pathology: Need to Redefine Bronchial Dilatation. Chest.

[12] Chang A.B., Zacharasiewicz A., Goyal V., Boyd J., Alexopoulou E., Aliberti S., Bell L., Bush A., Claydon A., Constant C. (2022). Task Force Report: European Respiratory Society Statement for Defining Respiratory Exacerbations in Children and Adolescents with Bronchiectasis for Clinical Trials. Eur. Respir. J..

[13] Zhao C., Tang H., McGonigle D., He Z., Zhang C., Wang Y.P., Deng H.-W., Bober R., Zhou W. (2021). A New Approach to Extracting Coronary Arteries and Detecting Stenosis in Invasive Coronary Angiograms. arXiv.

[14] Chang A.B., Masel J.P., Boyce N.C., Wheaton G., Torzillo P.J. (2003). Non-CF Bronchiectasis: Clinical and HRCT Evaluation. Pediatr. Pulmonol..

[15] Bhalla M., Turcios N., Aponte V., Jenkins M., Leitman B.S., McCauley D.I., Naidich D.P. (1991). Cystic Fibrosis: Scoring System with Thin-Section CT. Radiology.

[16] Bedi P., Chalmers J.D., Goeminne P.C., Mai C., Saravanamuthu P., Velu P.P., Cartlidge M.K., Loebinger M.R., Jacob J., Kamal F. (2018). The BRICS (Bronchiectasis Radiologically Indexed CT Score): A Multicenter Study Score for Use in Idiopathic and Postinfective Bronchiectasis. Chest.

[17] Prasad M., Sowmya A., Wilson P. (2008). Automatic Detection of Bronchial Dilatation in HRCT Lung Images. J. Digit. Imaging.

[18] Barral M., Sirol M., El Hajjam M., Zhang N., Petit A., Cornelis F.H. (2020). Bronchial Artery Embolization Performed in COVID-19 Patients: Tolerance and Outcomes. Cardiovasc. Intervent. Radiol..

[19] Nardelli P., Jimenez-Carretero D., Bermejo-Pelaez D., Washko G.R., Rahaghi F.N., Ledesma-Carbayo M.J., San Jose Estepar R. (2018). Pulmonary Artery-Vein Classification in CT Images Using Deep Learning. IEEE Trans. Med. Imaging.

[20] Zhou C., Chan H.P., Sahiner B., Hadjiiski L.M., Chughtai A., Patel S., Wei J., Ge J., Cascade P.N., Kazerooni E.A. (2007). Automatic Multiscale Enhancement and Segmentation of Pulmonary Vessels in CT Pulmonary Angiography Images for CAD Applications. Med. Phys..

[21] Hefeda M.M. (2020). CT Chest Findings in Patients Infected with COVID-19: Review of Literature. Egypt. J. Radiol. Nucl. Med..

[22] Gu Q., Qi S., Yue Y., Shen J., Zhang B., Sun W., Qian W., Islam M.S., Saha S.C., Wu J. (2019). Structural and Functional Alterations of the Tracheobronchial Tree after Left Upper Pulmonary Lobectomy for Lung Cancer. Biomed. Eng. Online.

[23] Large COVID-19 CT Scan Slice Dataset|Kaggle. https://www.kaggle.com/datasets/maedemaftouni/large-covid19-ct-slice-dataset.

[24] Kuo W., Perez-Rovira A., Tiddens H., de Bruijne M., Akesson L., Bertolo S., Brody A.S., de Boeck K., de Jong P.A., Fleck R.J. (2020). Airway Tapering: An Objective Image Biomarker for Bronchiectasis. Eur. Radiol..

[25] Charbonnier J.P., Pompe E., Moore C., Humphries S., van Ginneken B., Make B., Regan E., Crapo J.D., van Rikxoort E.M., Lynch D.A. (2019). Airway Wall Thickening on CT: Relation to Smoking Status and Severity of COPD. Respir. Med..

[26] Li K., Wu J., Wu F., Guo D., Chen L., Fang Z., Li C. (2020). The Clinical and Chest CT Features Associated With Severe and Critical COVID-19 Pneumonia. Invest. Radiol..

[27] Qu H., Zhang W., Yang J., Jia S., Wang G. (2018). The Value of the Air Bronchogram Sign on CT Image in the Identification of Different Solitary Pulmonary Consolidation Lesions. Medicine.

[28] Liu: A Observational Autopsy Report of COVID-19 and—Google Scholar. https://scholar.google.com/scholar_lookup?journal=J+Forensic+Med&title=A+observational+autopsy+report+of+COVID-19+and+at+follow-up&author=XX+Liu&author=Q+Guoqiang&author=Y+Wang&volume=36&publication_year=2020&pages=19-21&.

[29] Kim V., Dolliver W.R., Nath H.P., Grumley S.A., Terry N., Ahmed A., Yen A., Jacobs K., Kligerman S., Diaz A.A. (2021). Mucus Plugging on Computed Tomography and Chronic Bronchitis in Chronic Obstructive Pulmonary Disease. Respir. Res..

[30] Carotti M., Salaffi F., Sarzi-Puttini P., Agostini A., Borgheresi A., Minorati D., Galli M., Marotto D., Giovagnoni A. (2020). Chest CT Features of Coronavirus Disease 2019 (COVID-19) Pneumonia: Key Points for Radiologists. Radiol. Medica.

[31] Duran J., Coll B., Sbert C. (2013). Chambolle’s Projection Algorithm for Total Variation Denoising. Image Process. Line.

[32] Yousefi J. (2011). Image Binarization Using Otsu Thresholding Algorithm. Ont. Can. Univ. Guelph.

[33] Manders E.M.M., Strackee J., Aten J.A. (1996). Largest Contour Segmentation: A Tool for the Localization of Spots in Confocal Images. Cytom. J. Int. Soc. Anal. Cytol..

[34] Rafid A.R.H., Azam S., Montaha S., Karim A., Fahim K.U., Hasan M.Z. (2022). An Effective Ensemble Machine Learning Approach to Classify Breast Cancer Based on Feature Selection and Lesion Segmentation Using Preprocessed Mammograms. Biology.

[35] Kumar B., Tiwari U.K., Kumar S., Tomer V., Kalra J. (2020). Comparison and Performance Evaluation of Boundary Fill and Flood Fill Algorithm. Int. J. Innov. Technol. Explor. Eng..

[36] BahadarKhan K., Khaliq A.A., Shahid M. (2016). A Morphological Hessian Based Approach for Retinal Blood Vessels Segmentation and Denoising Using Region Based Otsu Thresholding. PLoS ONE.

[37] Tang J. A Color Image Segmentation Algorithm Based on Region Growing. Proceedings of the 2010 2nd International Conference on Computer Engineering and Technology.

[38] Burney S.A., Tariq H. (2014). K-Means Cluster Analysis for Image Segmentation. Int. J. Comput. Appl..

[39] Lei X., Pan H., Huang X. (2019). A Dilated CNN Model for Image Classification. IEEE Access.

[40] Van Rikxoort E.M., Van Ginneken B. (2013). Automated Segmentation of Pulmonary Structures in Thoracic Computed Tomography Scans: A Review. Phys. Med. Biol..

[41] Vallabhaneni R.B., Rajesh V. (2018). Brain Tumour Detection Using Mean Shift Clustering and GLCM Features with Edge Adaptive Total Variation Denoising Technique. Alex. Eng. J..

[42] Dutta S., Dey G., Chakraborty S., Roy P., Dey N., Ray R. Adaptive Thresholding: A Comparative Study. Proceedings of the 2014 International Conference on Control, Instrumentation, Communication and Computational Technologies (ICCICCT).

[43] Salima A., Herdiyeni Y., Douady S. Leaf Vein Segmentation of Medicinal Plant Using Hessian Matrix. Proceedings of the 2015 International Conference on Advanced Computer Science and Information Systems (ICACSIS).

[44] Khalid N.E.A., Ibrahim S., Manaf M., Ngah U.K. Seed-Based Region Growing Study for Brain Abnormalities Segmentation. Proceedings of the 2010 International Symposium on Information Technology.

[45] Pérez J.M.M., Pascau J. (2013). Image Processing with ImageJ.

[46] Tatiraju S., Mehta A. (2008). Image Segmentation Using K-Means Clustering, EM and Normalized Cuts. Dep. EECS.

[47] Montaha S., Azam S., Rakibul Haque Rafid A.K.M., Islam S., Ghosh P., Jonkman M. (2022). A Shallow Deep Learning Approach to Classify Skin Cancer Using Down-Scaling Method to Minimize Time and Space Complexity. PLoS ONE.

[48] Montaha S., Azam S., Rafid A.R.H., Hasan M.Z., Karim A., Islam A. (2022). TimeDistributed-CNN-LSTM: A Hybrid Approach Combining CNN and LSTM to Classify Brain Tumor on 3D MRI Scans Performing Ablation Study. IEEE Access.

[49] Islam M.Z., Islam M.M., Asraf A. (2020). A Combined Deep CNN-LSTM Network for the Detection of Novel Coronavirus (COVID-19) Using X-Ray Images. Inform. Med. Unlocked.

[50] Li J., Jin K., Zhou D., Kubota N., Ju Z. (2020). Attention Mechanism-Based CNN for Facial Expression Recognition. Neurocomputing.

[51] Aditi M.K., Poovammal E. (2019). Image Classification Using a Hybrid Lstm-Cnn Deep Neural Network. Int. J. Eng. Adv. Technol..

[52] Hamdi S., Oussalah M., Moussaoui A., Saidi M. (2022). Attention-Based Hybrid CNN-LSTM and Spectral Data Augmentation for COVID-19 Diagnosis from Cough Sound. J. Intell. Inf. Syst..

[53] Hirra I., Ahmad M., Hussain A., Ashraf M.U., Saeed I.A., Qadri S.F., Alghamdi A.M., Alfakeeh A.S. (2021). Breast Cancer Classification from Histopathological Images Using Patch-Based Deep Learning Modeling. IEEE Access.

[54] Wei L., Ding K., Hu H. (2020). Automatic Skin Cancer Detection in Dermoscopy Images Based on Ensemble Lightweight Deep Learning Network. IEEE Access.

[55] Yaqub M., Feng J., Sultan Zia M., Arshid K., Jia K., Ur Rehman Z., Mehmood A. (2020). State-of-the-Art CNN Optimizer for Brain Tumor Segmentation in Magnetic Resonance Images. Brain Sci..

[56] Montaha S., Azam S., Rakibul Haque Rafid A.K.M., Ghosh P., Hasan M.Z., Jonkman M., De Boer F. (2021). BreastNet18: A High Accuracy Fine-Tuned VGG16 Model Evaluated Using Ablation Study for Diagnosing Breast Cancer from Enhanced Mammography Images. Biology.

